# The N-Terminal Propeptide of Type III Procollagen in Patients with Acute Coronary Syndrome: A Link between Left Ventricular End-Diastolic Pressure and Cardiovascular Events

**DOI:** 10.1371/journal.pone.0114097

**Published:** 2015-01-05

**Authors:** Cheng-Hung Lee, Wen-Chen Lee, Shang-Hung Chang, Ming-Shien Wen, Kuo-Chun Hung

**Affiliations:** Division of Cardiology, Department of Internal Medicine, Chang Gung Memorial Hospital, Linkou, Chang Gung University College of Medicine, Tao-Yuan, Taiwan; University of Louisville, United States of America

## Abstract

**Background:**

Despite the usefulness of N-terminal propeptide of type III procollagen (PIIINP) in detecting enhanced collagen turnover in patients with congestive heart failure, the value added by PIIINP to the use of clinical variables and echocardiography in relation to directly measured left ventricular (LV) end-diastolic pressure (EDP) and the outcome of acute coronary syndrome (ACS) has not been clearly defined.

**Methods and Results:**

This study involved 168 adult patients with ACS, who underwent echocardiography, measurement of serum PIIINP levels, and cardiac catheterization. Pulsed wave tissue Doppler imaging (PWTDI), which revealed mean peak systolic (s′), early (e′), and late diastolic (a′) velocities, was carried out and the eas index of LV function was evaluated: e′/(a′×s′). The patients were divided into three study groups based on the degree of LVEDP – normal (<16 mmHg), intermediate (16–30 mmHg), and high (>30 mmHg) LVEDP. All patients were followed-up to determine cardiac-related death and revascularization. Patients with high LVEDP had significantly more PIIINP than those with intermediate or normal LVEDP (all *post hoc p*<0.05). The presence of coronary artery disease, the left atrial volume index (LAVI), the ratio of transmitral early and late diastolic flow velocities, a′, and the eas index were significantly correlated with LVEDP. According to multiple stepwise analysis, PIIINP, LAVI and the eas index were the three independent predictors of the level of LVEDP (PIIINP, *p* <0.001; LAVI, *p* = 0.007; eas index, *p = *0.021). During follow-up (median, 24 months), 32 participants suffered from cardiac events, PIIINP and LAVI were significant predictors of cardiac mortality and hospitalization (PIIINP, hazard ratio (HR) 2.589, *p* = 0.002; LAVI, HR 1.040, *p* = 0.027).

**Conclusions:**

PIIINP is a highly effective means to evaluate LVEDP in patients with ACS. The PIIINP is also correlated with cardiac mortality and revascularization, providing an additional means of evaluating and managing patients with ACS.

## Introduction

Acute coronary syndromes (ACS) refer to a continuum from unstable angina to non-ST-elevation and ST-elevation myocardial infarction (MI). The prognosis of patients with ACS typically depends on the occurrence and extent of myocardial necrosis [1], [2]. Left ventricular end-diastolic pressure (LVEDP) is correlated strongly with myocardial damage and widely utilized to evaluate the prognosis of patients with ACS [3], [4], [5]; it is often measured during left heart catheterization [6], [7]. As is well known, elevated LVEDP reflects reduced global ventricular compliance because of LV stiffness and considerable filling pressure. Ischemia with subsequent impairment of myocardial contractility is related to increased LV filling pressure that is caused by an upward shift of the EDP point in the pressure–volume (PV) loop [8]. Therefore, high LVEDP that results from ischemia and the reversibility of filling pressure following revascularization is predictable, and it is responsible for various outcomes [9], [10]. Many parameters for the indirect assessment of LVEDP have been used, minimizing possible complications including vascular injury and such rare but severe effects as MI or stroke [11], but the non-invasive use is somewhat limited by image quality, heart rate, and endocardial border definition [12], [13], [14], [15], [16].

Myocardial histological changes, including extracellular collagen deposition, strongly influence LV systolic and diastolic properties. In patients with ischemic heart disease, hormonal and immune activations are considered to affect the progression of LV dysfunction and heart failure [17]. The N-terminal propeptide of type III procollagen (PIIINP) is an extension peptide of procollagen type III, which is cleaved off stoichiometrically during conversion from type III procollagen to type III collagen [18]. Elevated PIIINP reflects myocardial remodeling, which is associated with significant LV dilatation and a persistently depressed LV ejection fraction. [19] PIIINP is also associated with a poor prognosis of patients following acute myocardial infarction and dilated cardiomyopathy [13], [20]. The correlation between the level of PIIINP, based on direct information about LVEDP and the prognosis of patients with ACS remains seldom addressed [19], [20].

This study determines whether PIIINP concentration may be associated with ventricular compliance dysfunction in patients with ACS. We hypothesize that patient with a higher PIIINP level exhibit a higher LVEDP, which can be directly measured by catheterization, and a poorer prognosis.

## Methods

### Study Population

**Figure 1:**
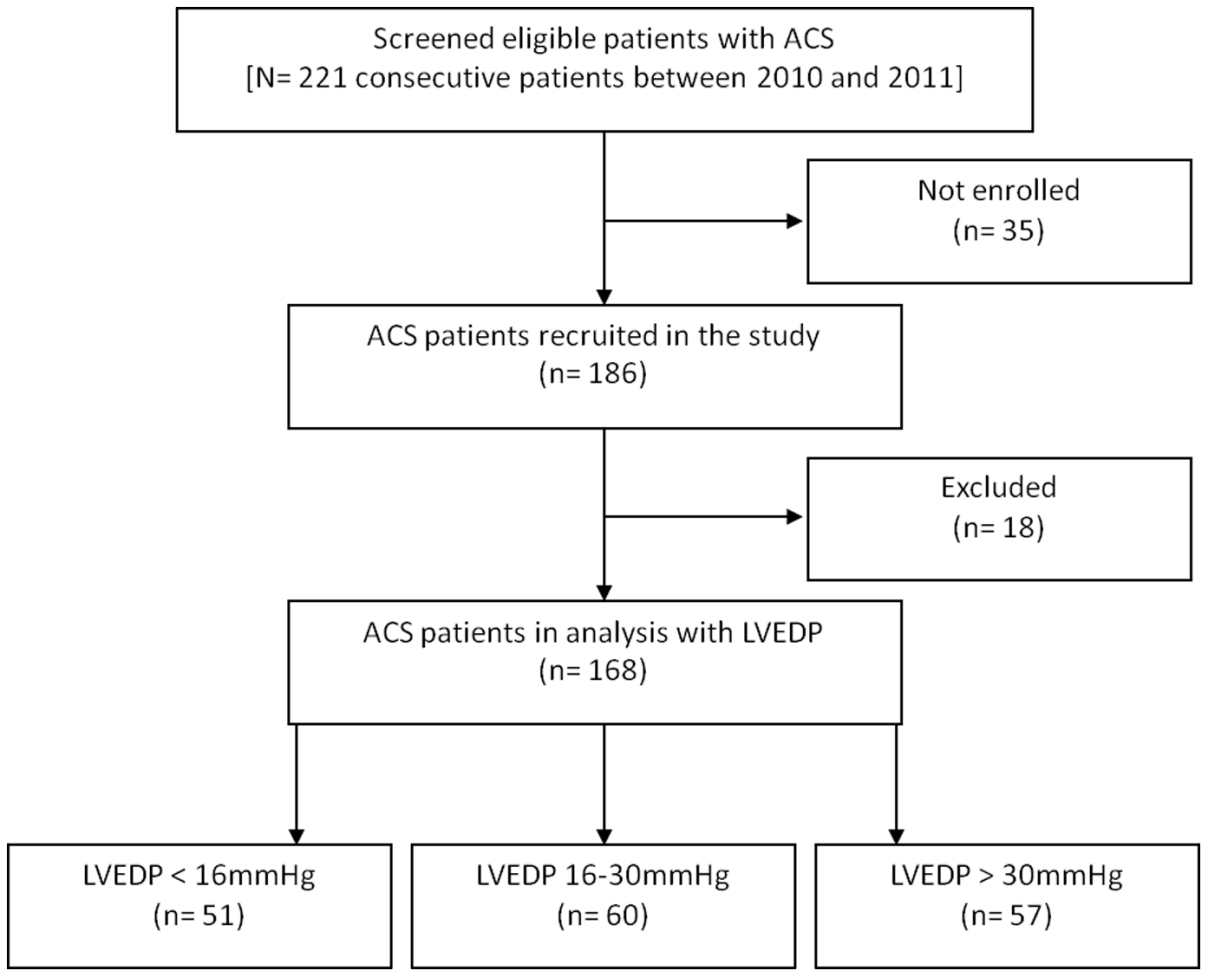
The flow chart of patient selection process.

The prospective investigation enrolled adult patients who had suffered their first ACS and were admitted to our hospital between 2010 and 2011 and scheduled to undergo percutaneous coronary intervention. Coronary wall atheromatous plaque with luminal reduction of greater than 50% will limit compensatory vasodilatation and thereby impede the flow coronary artery. Accordingly, coronary artery disease (CAD) is regarded as present when an obstruction of the vessel lumen exceeds 50%, as described elsewhere [21], [22]. LVEDP was recorded immediately before contrast injection. The highest LVEDP that was measured herein during the coronary catheterization procedure was recorded. The inclusion criteria of ACS are herein presentation within 24hours of an episode of ischemic chest pain (>10 minutes), either transient ST-segment elevation or depression (>0.05 mV), and a creatine kinase-MB fraction above the normal range. Patients with atrial fibrillation, significant valvular or congenital heart disease [23], [24], [25], or tissue fibrosis disease, such as chronic liver disease, pulmonary fibrosis, or rheumatoid arthritis [26], [27] were excluded, because these conditions are known to be associated with increased concentrations of PIIINP. Eighteen patients were excluded owing to liver disease (ten patients) and previous myocardial infarction (eight patients), leaving 168 patients (90%) for analysis (Fig. 1). Patients were categorized into three groups according to their LVEDP: (1) group A – normal, LVEDP <16 mmHg; (2) group B – intermediate, LVEDP between 16 and 30 mmHg; (3) group C – high, LVEDP greater than 30 mmHg [28], [29]. The investigation protocol was reviewed and approved by the institutional review board of Chang Gung Medical Foundation. Informed written consent was obtained from each patient before enrollment. The study was performed following the rules of the Helsinki Declaration.

### Laboratory Analyses

Serum samples for the measurement PIIINP concentrations were obtained before coronary catheterization. The concentration of PIIINP antigens in serum was evaluated by an equilibrium-type radioimmunoassay (Orion Diagnostica, Finland). Interassay and intra-assay variations for the PIIINP analyses were less than 7 %.

### Conventional M-mode, Two-dimensional (2D) and Doppler Echocardiography

Each subject underwent transthoracic M-mode, 2D and Doppler echocardiography using commercially available echocardiography units (Vivid 7, GE, U.S.A.) before cardiac catheterization was performed. The left atrium volume index (LAVI), the LV end-diastolic volume index (LVEDVI), the LV end-systolic volume index (LVESVI), and LVEF were evaluated in apical two - and four -chamber views using modified Simpson’s rule. Transmitral early (E) and late (A) diastolic flow velocities, the isovolumic relaxation time (IVRT), and the myocardial performance index (MPI) were also measured.

### Pulsed Wave Tissue Doppler Imaging (PWTDI)

A TDI of the mitral annulus was obtained at six sites with the apical four -chamber, two-chamber and long-axis views. The PWTDI, which incorporates the mean peak systolic (s′), early (e′), and late diastolic (a′) velocities at six mitral annular sites was evaluated. PWTDI was also calculated to generate a combined index (eas index) of LV performance: e′/(a′×s′). A value that combined transmitral flow velocity and annular velocity (E/e′) was estimated to quantify the LV filling pressure [30]. The velocity time integral that was obtained using PWTDI in the expiration phase was recorded and stored on videotape, digitalized and transferred to a digital-video disc for off-line analysis. At least three end-expiratory beats were evaluated, with their mean values were obtained.

### End Point and Follow-up

Follow-up information was obtained from clinical records, death certificates, and correspondence. The PIIINP and LVEDP values blinded for the physician who followed-up these patients. The clinical end-points of follow-up were cardiac death and re-hospitalization for ACS.

### Statistical Analysis

Continuous variables are expressed as mean ± SD, and categorical variables are expressed as absolute numbers (percentages). Unpaired *t*-test (continuous variables normally distributed), or Mann-Whitney U test (continuous variables which violated normal distribution assumption), and chi-squire test (categorical variables) were tested for the difference of clinical characteristics. For the comparison of continuous variables among multiple groups, one-way analysis of variance (ANOVA) and LSD post hoc test was used.

Multivariate association between LVEDP and the other parameters was determined by stepwise multiple linear regressions with variables that reached significance (*p* <0.1) in the preceding, unadjusted analysis of covariates of the 168 patients. Unadjusted Cox proportional hazards were used to evaluate the significance of various variables as predictors of cardiac death or re-hospitalization. Variables that were predictive of outcome (*p*<0.1) were then input into multivariate Cox proportional hazards regression models (backward selection) to identify independent predictors of outcomes. The outputs of the Cox regression analysis are presented as hazard ratios (HR) with a 95% confidence interval (CI). Cumulative curves for cardiac events were obtained using the Kaplan-Meier method. PIIINP concentrations were adjusted for age, baseline LVEF, gender, hypertension, and body mass index [31]. A *p* ≤ 0.05 was considered to indicate statistical significance. SPSS software (version 17.0 for Windows; SPSS Inc, Chicago, Illinois, USA) was utilized to analyze data.

## Results

Three patients died of cardiac causes; 24 patients were hospitalized for coronary revascularization, and five patients received coronary artery bypass therapy during a median follow-up period of 24 months.

### Patient Characteristics (Table 1)

The clinical characteristics of the cohort of 168 patients (113 men and 55 women) were analyzed. Fifty-one patients had normal LVEDP (<16 mmHg): 60 had intermediate LVEDP (between 16 and 30 mmHg), and 57 had high LVEDP (> 30 mmHg). The three groups resembled each other in age, male gender, heart rate, mean blood pressure, Killip class III or IV, hyperlipidemia, diabetes mellitus, and hypertension. Notably, group C contained a significantly higher percentage of patients with CAD than did in group A and B. The patients took the following medications; 145, antiplatelet agents; 83, angiotensin-converting enzyme inhibitors or angiotensin II receptor blockers; 133, beta-adrenoreceptor blockers; 76, statin. Ninety-three patients received coronary revascularization on initial management for ACS.

**Table 1:** Clinical Characteristics.

	Group A (n = 51)	Group B (n = 60)	Group C (n = 57)	*p* Values
Age(years)	60±11	61±14	58±13	0.374
Male Gender, %	32(63)	39(65)	42(74)	0.432
Heart rate	66±7	67±9	70±15	0.104
Mean BP (mmHg)	106±11	106±13	110±11	0.118
Coronary artery disease, %	29(57)	40(67)	51(90)	0.001† ‡
Killip III or IV, %	5(10)	14(23)	15(26)	0.078
Hyperlipidemia, %	18(35)	23(38)	29(51)	0.211
Diabetes mellitus, %	22(43)	29(48)	23(40)	0.677
Hypertension, %	27(53)	30(50)	24(42)	0.500
**Medications**				
Antiplatelet agents, %	43(84)	51(85)	51(90)	0.690
ACEI or ARB, %	25(49)	32(53)	26(46)	0.704
Beta blockers, %	39(77)	49(82)	45(79)	0.797
Statin, %	18(35)	32(53)	26(46)	0.163
**Therapy**				
Coronary revascularization, %	26(51)	34(57)	33(58)	0.746

† Group A (LVEDP <16 mmHg) versus Group C (LVEDP> 30 mmHg), *p*<0.05 in *post hoc* analysis.

‡ Group B (LVEDP 16–30 mmHg) versus Group C, *p*<0.05 in *post hoc* analysis.

ACEI, angiotensin converting enzyme inhibitor; ARB, angiotensin II receptor blocker; BP, blood pressure; LVEDP, left ventricular end-diastolic pressure; NYHA, New York Heart Association.

### Conventional and PWTDI Echocardiography (Table 2)

Comparing the patients with high LVEDP (Group C) with those in group A or group B revealed that they significantly differed in LA volume index, E/A ratio, a′, and eas index.

**Table 2:** Conventional Two-dimensional and Doppler Echocardiographic Findings.

	Group A (n = 51)	Group B (n = 60)	Group C (n = 57)	*p* Values
**M-mode and 2-D**				
LA volume index (ml/mm^2^)	32.5±6.3	33.0±6.1	36.4±9.6	0.012† ‡
LVEDV index (ml/m^2^)	70.9±17.0	70.8±16.9	70.3±17.8	0.982
LVESV index (ml/m^2^)	26.3±13.4	23.8±10.2	26.0±14.3	0.501
LV mass index (g/m^2^)	137.9±49.1	130.8±30.8	131.4±26.5	0.538
LVEF (%)	64.7±12.6	67.0±8.1	64.6±10.8	0.395
MPI	0.59±0.20	0.47±0.26	0.40±0.36	0.232
**Mitral inflow velocities**				
E (cm/s)	70.0±13.7	71.8±21.1	76.8±21.1	0.155
A (cm/s)	79.0±17.4	75.3±20.8	70.7±22.3	0.105
E/A	0.94±0.29	1.04±0.47	1.27±0.81	0.008†
IVRT (ms)	96.7±18.4	92.2±11.5	89.9±19.7	0.106
**Tissue Doppler Imaging**				
s′ (cm/s)	7.3±0.6	7.4±0.4	7.3±0.5	0.401
e′ (cm/s)	5.6±1.1	5.9±1.9	6.3±2.5	0.435
a′ (cm/s)	9.3±1.1	9.0±1.2	8.4±1.7	0.039†
E/e′	11.4±2.8	12.3±3.6	13.2±5.3	0.308
eas index	0.08±0.02	0.09±0.05	0.11±0.04	0.041†

† Group A versus Group C, p<0.05 in post hoc analysis.

‡ Group B versus Group C, p<0.05 in post hoc analysis.

A, late diastolic transmitral Doppler flow velocity; a′, late mitral annular diastolic velocity; DT, deceleration time; E, early diastolic transmitral Doppler flow velocity; eas, ratio e′/(a′ X s′); EDV, end diastolic volume; EF, ejection fraction; ESV, end systolic volume; e′, early mitral annular diastolic velocity; IVRT, isovolumic relaxation time; LA, left atrium; LV, left ventricular; MPI, myocardial performance index; s′, mitral annular systolic velocity.

Patients with ACS in all three groups exhibit the same degree of LVEDVI, LVESVI, LV mass index, LVEF and MPI. Moreover, these patients in the three groups exhibited comparable E, A, IVRT, s′, e′, a′ and E/e′ ratio values across the three groups.

**Figure 2:**
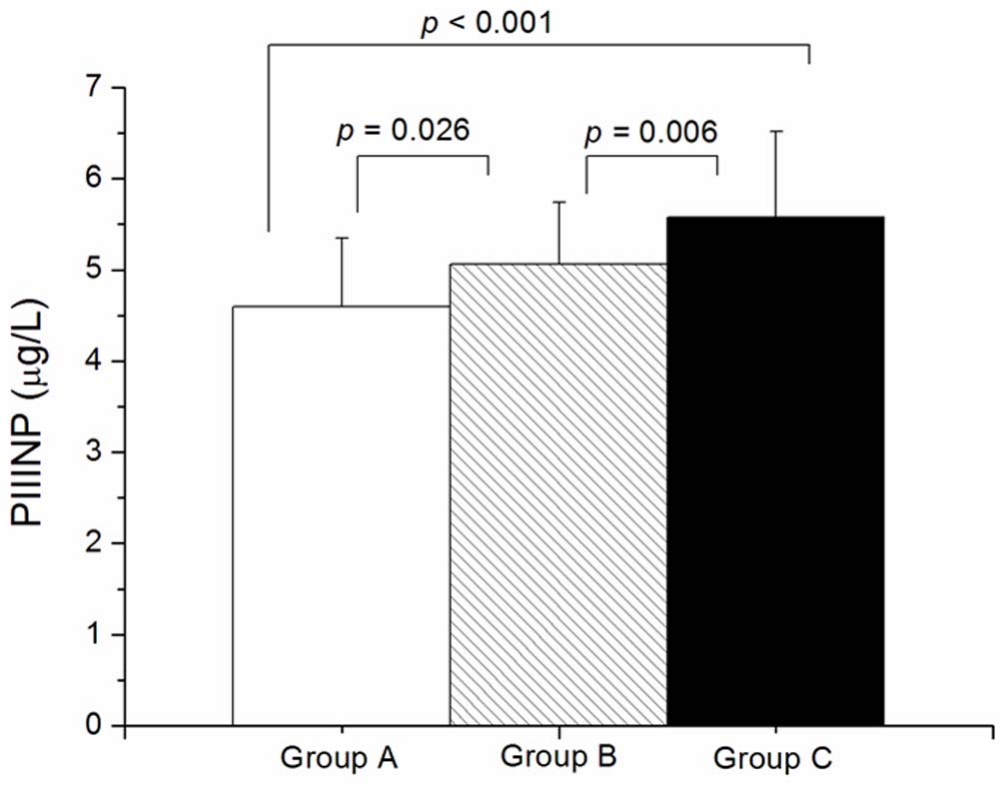
Concentrations of N-terminal propeptide of type III procollagen in three groups of patients with acute coronary syndrome.

As present in Fig. 2, serum PIIINP concentration varied significantly among groups, following adjustments for age, baseline LVEF, gender, hypertension, and body mass index. In particular, group C patients (5.58 ± 0.94 *µ*g/L) exhibited a higher concentration of PIIINP than both group A (4.60 ± 0.75 *µ*g/L) and group B (5.05 ± 0.68 *µ*g/L), and group B exhibited a higher concentration thereof than group A (*p* all <0.05).

### Relationship between PIIINP and LVEDP

When LVEDP was the outcome variable, and the presence of CAD, LAVI, E/A ratio, a′, eas index and PIIINP level were covariates. PIIINP concentration (standardized coefficients (r) = 0.373, *p* <0.001), LAVI (r = 2.752, *p* = 0.007) and eas index (r = 2.354, *p* = 0.021) were independently related to the other covariates on LVEDP.

### End point and Cardiac outcome

**Figure 3:**
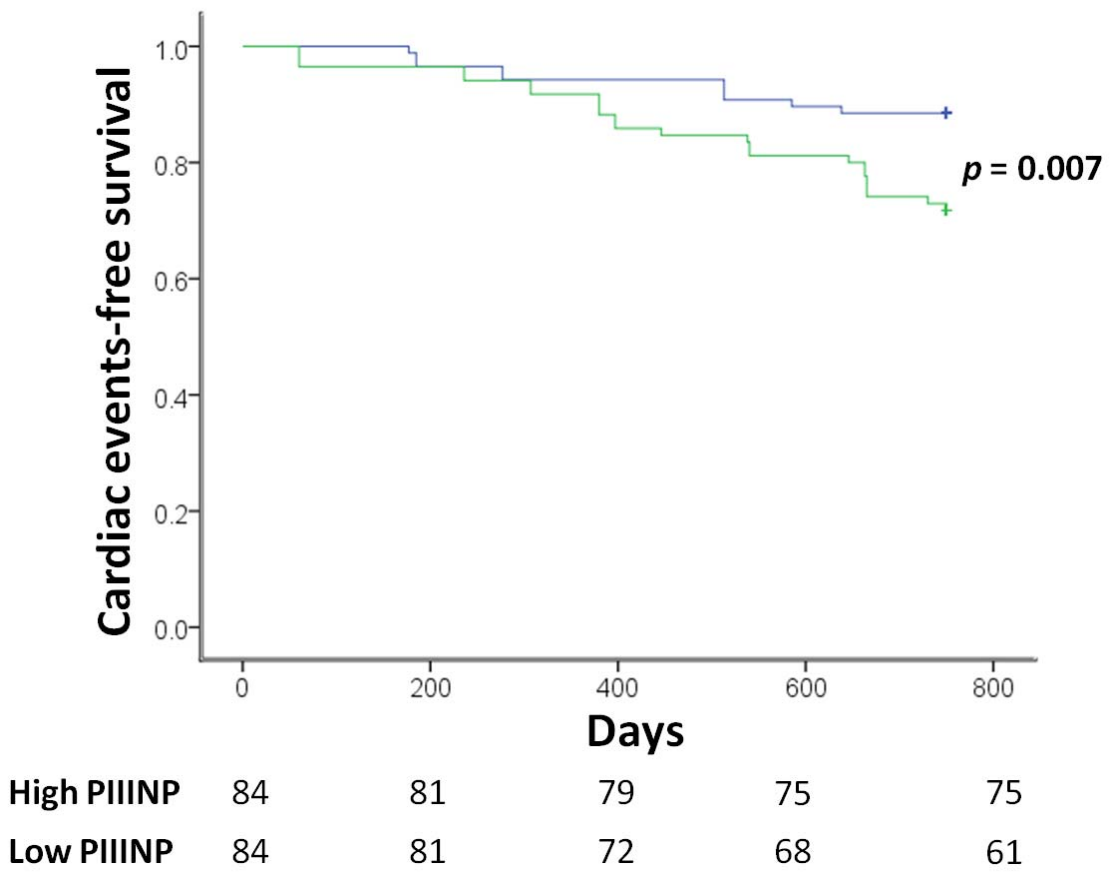
Cardiac events-free survival curves categorized by whether PIIINP level is higher (upper curve) or less (lower curve) than 5.09 (median value). Number of patients at risk during follow-up is indicated below the x axis.

The mortality and morbidity rates of the patients over 24 months were 19 %. All of the variables that predicted the combined outcome of death and hospitalization based on unadjusted Cox regression analysis (p<0.1) were input a backward multivariate Cox regression analysis. PIIINP and LAVI emerged as independent predictors of outcome for patients with ACS (PIIINP, HR 2.589, 95% CI, 1.404–4.773, *p* = 0.002; LAVI, HR 1.040, 95% CI, 1.005–1.076, *p* = 0.027). Table 3 presents the final multivariate Cox model. Figure 3 plots the Kaplan-Meier curves of the patients with ACS, categorized by whether PIIINP is greater or less than 5.09 (median value). As expected, the patient group the mean of higher than 5.09 had the worse outcomes (*p* = 0.007; log-rank test).

**Table 3:** Unadjusted and multivariate predictors of cardiac death and hospitalization.

	Unadjusted analysis		Multivariate analysis	
	HR(95% CI)	*p* Value	HR(95% CI)	*p* Value
PIIINP*	2.116(1.147–3.323)	0.001	2.589(1.404–4.773)	0.002
LA volume index	1.039(1.005–1.074)	0.025	1.040(1.005–1.076)	0.027
Coronary artery disease	2.018(1.543–2.641)	0.006	NA	NA
Hyperlipidemia	1.791(0.903–3.555)	0.096	NA	NA
LVEF	0.076(0.004–1.288)	0.074	NA	NA
s′	0.641(0.471–0.872)	0.005	NA	NA
E/e′	1.091(1.005–1.184)	0.038	NA	NA

CI, confidence interval; HR, hazard ratio; NA, not applicable.

*adjusted for age, baseline LVEF, gender, hypertension, and body mass index.

## Discussion

This investigation studies the association of LVEDP with clinical manifestations in patients with ACS, and reveals that PIIINP concentration is an independent predictor of LVEDP. The PIIINP concentration also correlates closely with cardiac mortality and hospitalization for revascularization.

### LVEDP and CAD

The pathophysiology that underlies high LVEDP is strongly related to stiffness of the LV chamber [32], which reduces end-diastolic volumes and thereby reduces stroke volumes, as revealed by the shift to the left and narrowing of the LV PV loops [33]. Moreover, the non-linearity of the PV curve indicates increased diastolic pressure upon LV remodeling. The histological characteristics of the myocardium affect the position and the slope of the curve [34]. Accordingly, LVEDP becomes elevated because of increased myocardial fibrosis, cardiomyocyte hypertrophy, or alteration of the myocardial microvascular structure following CAD-related ischemic cardiomyopathy [35]. Hence, a high proportion of patients with CAD exhibited elevated LVEDP in this cohort study.

During ventricular diastole, the LA is directly exposed to LV pressure through the open mitral valve. LA size is therefore largely governed by the factors that affect diastolic LV filling [36]. The LA is a volume sensor, and the atrial wall releases natriuretic peptides in response to stretch-generating potent effects of increased LV filling pressure [37]. Extensive evidence has shown that an enlarged LA indicates significant ventricular, atrial, or valvular disease. Therefore, increased LA size is increasingly regarded as a predictor of poorer cardiovascular outcomes [38], [39]. These findings help to explain the increased LAVI in patients with ACS, which reflects elevated LV filling pressure herein.

### LVEDP, E/A ratio, a′, and eas index

The transmitral flow pattern and TDI remain the most effective means of routinely measuring systolic and diastolic function and predicting prognosis in patients with heart failure [30], [40]. Sub-endocardial ischemia is induced by elevated LV filling pressure. Hence, mitral annular velocity, determined using TDI, reflects the lengthening of LV myocardial fibers of the sub-endocardium in the longitudinal direction, and appears to be useful in assessing the consequences of ischemia [41]. A reduced a′ of mitral annular velocity also reflects elevated pulmonary venous pressure in patients with LV dysfunction [42]. Relevant investigations be consistent the echocardiographic findings herein that a high E/A ratio, reduced a′, and elevated eas index provide valuable information for classifying patients that are diagnosed with ischemic heart disease.

### LVEDP, PIIINP, and cardiovascular events

PIIINP is regarded as a circulating collagen biomarker of extracellular matrix remodeling in the heart because elevated PIIINP concentration reflects increased collagen turnover, involving synthesis and deposition, as well as alternating degradation and elimination [43], [44]. PIIINP levels in this investigation were positively correlated with the measured LVEDP. We hypothesize that this finding follows from an increase in collagen turnover that is caused by more aggressive ventricular remodeling in patients with greater LV filling pressure. This suggestion is supported by the finding that PIIINP levels were higher in patients with elevated LVEDP and by the fact that PIIINP level was correlated positively with levels of markers of worsening cardiovascular outcome [45]. Interestingly, PIIINP levels were positively correlated with LVEDP levels, suggesting that PIIINP may also be a potential indicator of disease severity. Furthermore, a correlation between higher PIIINP levels and the worsening of the disease was identified, suggesting that the circulating biomarker may reflect ongoing vascular and ventricular remodeling.

Numerous studies have claimed that increased PIIINP is related to a restrictive mitral filling pattern and diastolic dysfunction [46], [47], [48]. However, few data are available on the relationship between PIIINP levels and invasively and directly evaluated LVEDP. In this investigation, PIIINP level is an independent predictor of LVEDP. The use of PIIINP for monitoring would result in considerable savings in the healthcare budget and a possible reduction of complications [11], [49]. Cardiac mortality and hospitalization for revascularization in patients with ACS are predicted. Based on the results herein, we speculate that in patients with ischemic heart disease, levels of markers of cardiac fibrosis correlate strongly with LVEDP. Thus, the PIIINP level may add significant value to the clinical follow-up and management of patients with ischemic-related myocardial damage.

### Limitations of Study

Despite its contributions, this investigation has some limitations. The number of participating patients was still relatively small and a larger patient population should be analyzed to verify the analytic results herein. Additionally, patients with ACS in this cohort study may not be homogeneous comparing with previous studies including the percentage of revascularization as well as administration of beta blockers [50], [51]. Moreover, the single measurement of PIIINP concentration may be a limitation of this study. Finally, patients with atrial fibrillation and significant valvular heart disease were excluded, so a substantial number of patients with ACS were excluded, possibly generating a bias in the analytical results.

### Conclusions and Clinical Implications

The evaluation of cardiac fibrosis using PIIINP level may contribute significantly to efforts to evaluate and manage patients with unstable angina, non-ST-elevation or ST-elevation MI. The PIIINP level can be used to distinguish patients with normal LVEDP from those with intermediate or high LVEDP, supporting the importance of LVEDP in the pathophysiological mechanism of cardiac fibrosis in these patients. The PIIINP level also predicts clinical outcome.
